# A Case of Esophageal Penetration of a Fish Bone into the Left Atrium Rescued by Urgent Surgery

**DOI:** 10.70352/scrj.cr.25-0445

**Published:** 2025-10-31

**Authors:** Norihiko Sakamoto, Shinsuke Takeno, Makoto Ikenoue, Fumiaki Kawano, Shun Munakata, Ikko Shimizu, Risako Kojima, Risa Meiri, Ayaka Iwasaki, Kousuke Mori, Shuhei Sakaguchi, Hirohito Ishii, Koji Furukawa, Atsushi Nanashima

**Affiliations:** Department of Surgery, Faculty of Medicine, Miyazaki University, Miyazaki-city, Miyazaki, Japan

**Keywords:** fish bone, esophageal penetration, left atrium penetration

## Abstract

**INTRODUCTION:**

Esophageal penetration due to accidental foreign body ingestion is relatively rare but has a poor prognosis. Penetration into the left atrium is extremely rare, and only a few cases have been reported. A case of foreign body penetration into the left atrium is reported.

**CASE PRESENTATION:**

The patient was a 75-year-old woman who was transferred to our hospital for surgery due to fish bone perforation from the lower esophagus into the left atrium on CT. Urgent surgery was performed with the diagnosis of sepsis due to a mediastinal abscess and esophageal penetration into the left atrium caused by accidental fish bone ingestion. At surgery, only the fistula of the penetration wound in the esophagus and the left atrium could be detected, but not the fish bone. On CT after surgery, a folded fish bone was seen at the pericardium close to the left atrium. It was considered unlikely that the heart would again be perforated due to the fish bone’s length, and it was decided to follow up with continued antimicrobial therapy.

**CONCLUSIONS:**

A rare, successfully rescued case of esophageal penetration of an accidentally ingested fish bone into the left atrium by urgent surgery, with collaboration between gastrointestinal and cardiovascular surgeons, is reported.

## INTRODUCTION

Esophageal penetration by foreign body ingestion, such as dentures, fish bones, and press-through packages, is relatively rare and has a poor prognosis that can lead to serious complications, such as mediastinitis, pyothorax, and sepsis. However, a case of penetration into the left atrium is extremely rare, and very few cases have been reported. A case of esophageal penetration of a fish bone into the left atrium rescued by urgent surgery is presented.

## CASE PRESENTATION

**Fig. 1:**
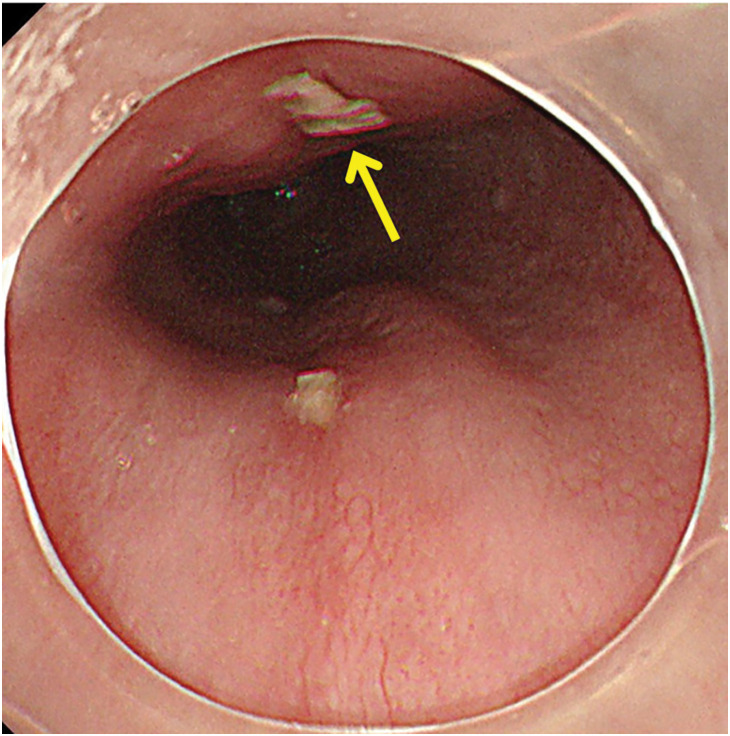
Images from upper gastrointestinal endoscopy performed at a previous hospital. The arrow indicates the penetration site on the ventral side of the esophagus, where a white object was observed, with a similar object also noted on the dorsal side.

A 75-year-old woman who had swallowed a snapper fish bone 2 days earlier visited the hospital with the chief complaint of high fever and persistent chest discomfort. CT showed fish bone penetration from the lower thoracic esophagus into the left atrium. An upper gastrointestinal endoscopy demonstrated a penetrating site on the ventral side of the esophagus, at which a white deposit was noted. A similar deposit was observed on the dorsal side, although the fish bone itself could not be identified (**Fig. 1**). These deposits were considered most likely to represent purulent exudates. The patient was transported to our hospital for urgent surgery.

**Fig. 2:**
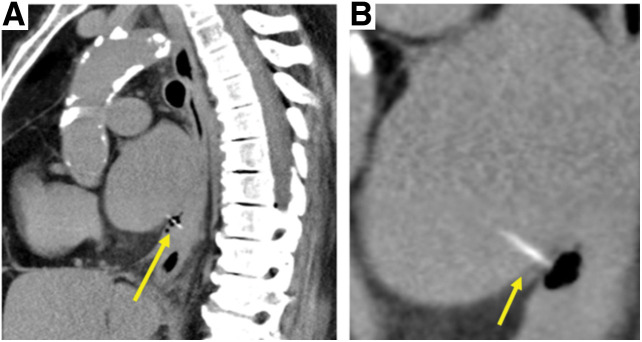
The first CT scan at our hospital. (**B**) is a magnified view of (**A**). A 2.5-cm-long, linear, hyperabsorptive structure penetrating the left atrium from the ventral side of the lower thoracic esophagus is observed (yellow arrows). Free air and fluid collection are also observed in the lower mediastinum.

On arrival at the hospital, fever, tachycardia, and decreased SpO_2_ were observed, and blood tests showed elevated white blood cell and C-reactive protein levels. CT showed a 2.5-cm-long, linear, hyperabsorptive zone penetrating from the ventral side of the lower esophagus of the chest to the left atrium (**Fig. 2**).

Urgent surgery was performed with the diagnosis of sepsis due to a mediastinal abscess and esophageal penetration of an accidentally ingested fish bone into the left atrium. The urgent surgery involved a thoracic esophagectomy and esophagostomy, an attempt to remove the fish bone, and the purification and drainage of a mediastinal abscess by the gastrointestinal and cardiovascular surgeons.

**Fig. 3:**
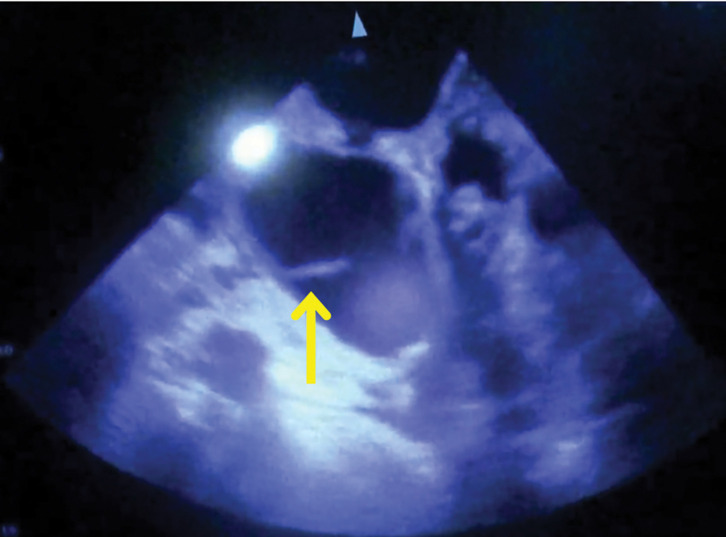
Intraoperative echocardiography. The arrow indicates the fish bone penetrating into the left atrium.

**Fig. 4:**
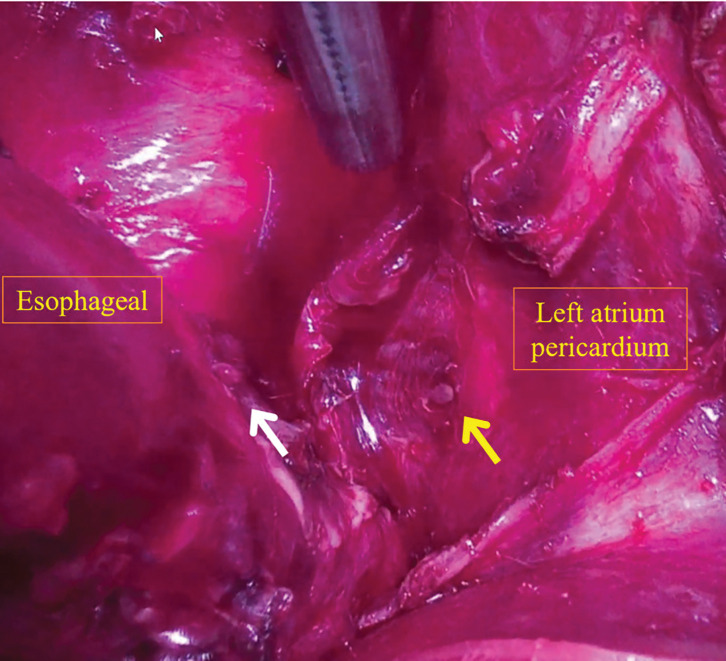
Intraoperative images. The esophagus is located on the left side of the image, and the pericardium of the left atrium is on the right. The white arrow indicates the site of esophageal perforation, while the yellow arrow shows the fistula entrance of the fish bone into the left atrium.

**Fig. 5:**
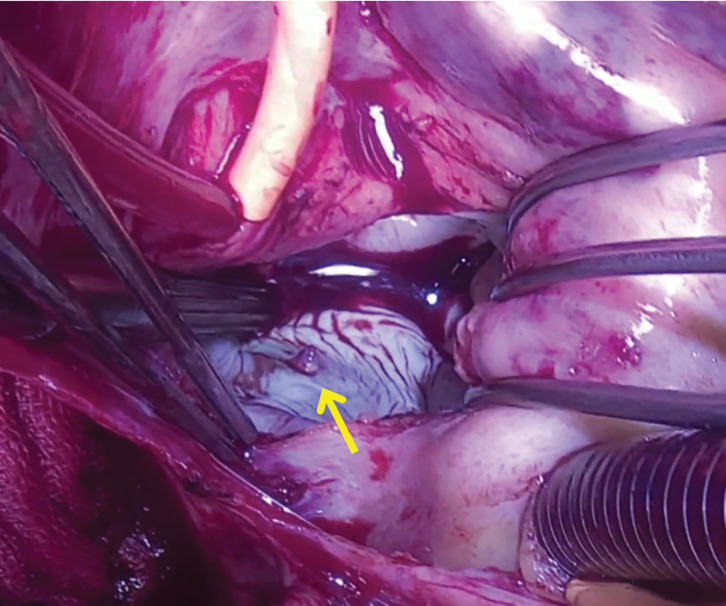
Intraoperative view after left atriotomy under the artificial heart–lung machine. The arrow indicates the fistula exit into the left atrium.

The surgery was started with the patient in the left decubitus position under general anesthesia with left 1-lung ventilation. During surgery, drainage of pus was observed from a pericardial abscess just above the diaphragm during dissection of the esophagus from the pericardial sac. An open chest echocardiogram again confirmed a fish bone penetrating into the left atrium (**Fig. 3**). Subsequently, cardiovascular surgeons checked the pericardium, and they found only a fistula entrance, but no fish bone (**Fig. 4**). Then, a search for the fish bone was conducted in the left atrium with the patient on the artificial heart–lung machine, but only the fistula exit, not the fish bone, could be found (**Fig. 5**). A further search for the fish bone was conducted, considering that the bone may have strayed into the cardiopulmonary circuit or adhered to the surgical gauze or the instruments, but it could not be located. The operation was terminated with closure of the fistula in the left atrium, thoracic esophagectomy, and cervical esophagostomy.

**Fig. 6:**
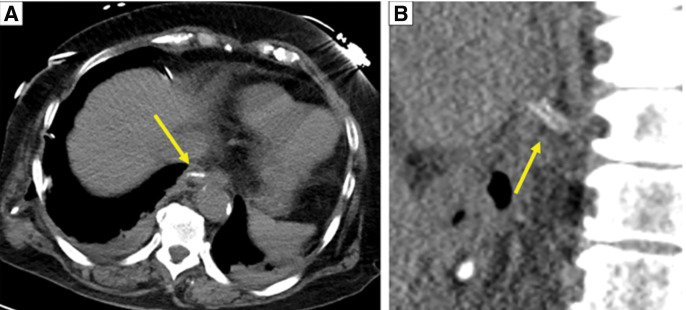
CT immediately after surgery. (**A**) Axial view and (**B**) sagittal view. A double-linear hyperabsorptive structure is observed near the esophagectomy site (yellow arrows).

An object folded in half was detected at the pericardium close to the left atrium on CT after the surgery. Its intensity was similar to the preoperative intensity of the fish bone (**Fig. 6**).

We thought that the fish bone had entered the left atrium from the esophagus and appeared to have been removed from the left atrium and folded in 2 during operative manipulation. We discussed the matter with cardiovascular surgeons and determined that the length of the fish bone was about 1 cm and that it was folded in half, so the possibility of re-perforation was low. Therefore, we chose conservative treatment.

During the patient’s postoperative course, left temporal lobe and right cerebellar infarctions appeared on POD 6. CT on PODs 9 and 24 showed no change in the structures that appeared to be the fish bone. The patient was transferred to the previous hospital for the purpose of rehabilitation on POD 38.

At post-operative month 16, although it was difficult to communicate with her due to the aftereffects of the cerebral infarction, her general condition was good, and there were no signs of infection. It was difficult to confirm her wishes, and her family could not decide on the reconstruction surgery, so it was postponed. After that, her family agreed to the surgery, and we were planning to perform the reconstruction surgery through the antethoracic route.

## DISCUSSION

Esophageal foreign bodies are occasionally encountered in emergency medicine. The majority (80%–90%) of esophageal foreign bodies pass spontaneously, but approximately 10%–20% of cases require endoscopic removal, and less than 1% require surgery to remove the foreign body or treat complications.^1)^

Esophageal foreign bodies rarely cause esophageal perforation. Without proper diagnosis and treatment, however, they can lead to death due to mediastinitis or sepsis. The mortality rate for esophageal perforation is 10%–20%.^2,3)^ Patients treated within 24 h of esophageal perforation have a mortality rate of less than 10%, compared with 30% for patients treated later.^4–6)^ Altorjay et al. provided criteria for nonoperative treatment of esophageal perforation.^7)^ In the present case, 24 h after onset, the patient was septic and had other organ damage, so surgical treatment was selected. Since 2 days had elapsed between onset and the hospital visit, it was assumed the patient had a poor prognosis. The fact that the patient was able to be rescued despite the poor prognosis is thought to be the result of appropriate measures taken at each point, such as immediate transport to a hospital where gastroenterological and cardiovascular surgeons were available after diagnosis, and appropriate surgical procedures such as abscess drainage.

Injury to the pericardium or great vessels by a foreign body can lead to serious complications such as hemorrhage, cardiac tamponade, and suppurative pericarditis.^8,9)^ Reports of cases involving perforation of the esophagus and atrium are very rare; a PubMed search using the keyword combination “esophageal perforation,” “fish bone,” and “atrium” identified 3 cases.^10–12)^ Reportedly, emergency surgery and endoscopic retrieval of the fish bone are life-saving. However, few of the reports described the procedure in detail, which made it difficult to select a technique in the present case. If we can easily identify the fish bone by dissecting the esophagus, we remove it directly and preserve the esophagus unless cardiac tamponade or similar issues are present. However, in this case where we observed the fistula but could not visualize the fish bone, we must resect the esophagus to ensure a safe surgical field and to manipulate the delicate heart. So, we performed an esophagectomy for securing the view of the field and removing the perforation site, and then checked the pericardium. Since we thought that esophagectomy would enable us to confirm the fish bone, it was unexpected that we could not confirm it on the surface of the pericardium. Although we chose this surgical approach to minimize the surgical invasion, we consider that if there are similar cases, we will have the option of using the artificial heart–lung machine early and performing a search in the atrium. Regarding the retention of a fish bone, a PubMed search using the keyword combination “fish bone” and “conservative treatment” identified only 1 case.^13)^ In their reported case, the authors were highly concerned that discontinuation of antiplatelet therapy would significantly increase the risk of recurrent cardiac events, given the patient’s recent myocardial infarction, severe triple-vessel disease, and recent coronary stent implantation. In our case, although the risk of re-perforation and infection cannot be ruled out, considering the risks associated with surgery, we chose conservative treatment.

## CONCLUSIONS

In an extremely rare case of esophageal penetration of a fish bone into the left atrium, the patient was rescued through emergency surgery by taking full advantage of our comprehensive surgical department.
